# Characterisation of the NRF2 transcriptional network and its response to chemical insult in primary human hepatocytes: implications for prediction of drug-induced liver injury

**DOI:** 10.1007/s00204-018-2354-1

**Published:** 2018-11-13

**Authors:** Ian M. Copple, Wouter den Hollander, Giulia Callegaro, Fiona E. Mutter, James L. Maggs, Amy L. Schofield, Lucille Rainbow, Yongxiang Fang, Jeffrey J. Sutherland, Ewa C. Ellis, Magnus Ingelman-Sundberg, Stephen W. Fenwick, Christopher E. Goldring, Bob van de Water, James L. Stevens, B. Kevin Park

**Affiliations:** 1 MRC Centre for Drug Safety Science, Department of Molecular and Clinical Pharmacology, Institute of Translational Medicine, University of Liverpool, Liverpool, L69 3GE UK; 2 Section of Pharmacogenetics, Department of Physiology and Pharmacology, Karolinska Institute, 171-77 Stockholm, Sweden; 3 Division of Toxicology, Leiden Academic Centre for Drug Research, Leiden University, 2333 CC Leiden, The Netherlands; 4 Centre for Genomic Research, Institute of Integrative Biology, University of Liverpool, Liverpool, L69 7ZB UK; 5 Lilly Research Laboratories, Eli Lilly and Company, Indianapolis, IN 46285 USA; 6 Liver Cell Lab, Unit for Transplantation Surgery, Department of Clinical Science, Intervention and Technology (CLINTEC), Karolinska University Hospital Huddinge, 141-86 Stockholm, Sweden; 7 Department of Hepatobiliary Surgery, Aintree University Hospital NHS Foundation Trust, Liverpool, L9 7AL UK

**Keywords:** DILI, NFE2L2, KEAP1, WGCNA, Oxidative stress

## Abstract

The transcription factor NRF2, governed by its repressor KEAP1, protects cells against oxidative stress. There is interest in modelling the NRF2 response to improve the prediction of clinical toxicities such as drug-induced liver injury (DILI). However, very little is known about the makeup of the NRF2 transcriptional network and its response to chemical perturbation in primary human hepatocytes (PHH), which are often used as a translational model for investigating DILI. Here, microarray analysis identified 108 transcripts (including several putative novel NRF2-regulated genes) that were both downregulated by siRNA targeting NRF2 and upregulated by siRNA targeting KEAP1 in PHH. Applying weighted gene co-expression network analysis (WGCNA) to transcriptomic data from the Open TG-GATES toxicogenomics repository (representing PHH exposed to 158 compounds) revealed four co-expressed gene sets or ‘modules’ enriched for these and other NRF2-associated genes. By classifying the 158 TG-GATES compounds based on published evidence, and employing the four modules as network perturbation metrics, we found that the activation of NRF2 is a very good indicator of the intrinsic biochemical reactivity of a compound (i.e. its propensity to cause direct chemical stress), with relatively high sensitivity, specificity, accuracy and positive/negative predictive values. We also found that NRF2 activation has lower sensitivity for the prediction of clinical DILI risk, although relatively high specificity and positive predictive values indicate that false positive detection rates are likely to be low in this setting. Underpinned by our comprehensive analysis, activation of the NRF2 network is one of several mechanism-based components that can be incorporated into holistic systems toxicology models to improve mechanistic understanding and preclinical prediction of DILI in man.

**Electronic supplementary material:**

The online version of this article (10.1007/s00204-018-2354-1) contains supplementary material, which is available to authorized users.

## Introduction

Drug-induced liver injury (DILI) remains a leading cause of clinical liver failure and a hindrance to the development of new therapies, making it a priority area for the advancement of new risk prediction methods (Chen et al. 2014). Hepatocellular perturbation is often coupled to the activation of stress-responsive transcription factors that regulate networks of cytoprotective genes. By reflecting activation of stress-inducible transcriptional networks, such genes are considered useful mechanistic markers of the cellular consequences of chemical insult (Wink et al. 2014). The increasing availability of public data sets describing transcriptional responses to large numbers of compounds provides an excellent opportunity to explore the behaviour of key stress-responsive networks from a broad perspective. One example of such a data set is the Japanese Toxicogenomics Project’s Open TG-GATES repository, which contains transcriptomic data from primary human hepatocytes (PHH) exposed to 158 compounds (primarily therapeutic drugs, but also experimental toxins, cytokines and growth factors) at up to three different concentrations and time points (Igarashi et al. 2015). This and other resources can support a deeper understanding of the molecular make-up of stress-inducible transcriptional networks, which is key to the design of, and interpretation of data from, preclinical assays and in silico models that can reliably reflect their perturbation, and thus provide a necessary level of confidence in the understanding and prediction of relevant human risk (Oshida et al. 2015a, b, c; Sutherland et al. 2018).

Chemical and oxidative stresses are associated with the adverse effects of many compounds, particularly electrophiles and free radicals that react with critical macromolecules and disrupt normal redox processes (Park et al. 2011; Pereira et al. 2012). In mammalian cells, these insults are counteracted by an antioxidant stress response directed by the transcription factor nuclear factor erythroid 2 related factor 2 (NRF2 or NFE2L2) (Bryan et al. 2013). Governed by its repressor Kelch-like ECH-associated protein 1 (KEAP1), NRF2 regulates the basal and inducible expression of cytoprotective genes which contain antioxidant response elements (AREs) in their promoter and/or enhancer regions (Bryan et al. 2013). Despite the mechanistic association of NRF2 with DILI and other forms of drug toxicity in rodents (Clarke et al. 2016), and the increasing interest in using translationally relevant human platforms to improve hazard prediction in man, relatively little is known about the makeup of the NRF2 transcriptional network and its response to chemical perturbation in PHH, which are often used as a translational in vitro model for investigating DILI. To address this knowledge gap, we have used siRNA to modulate NRF2 activity and identify genes that respond robustly to perturbation of the network in freshly-isolated PHH. Using an unbiased weighted gene co-expression network analysis (WGCNA) approach (Langfelder and Horvath 2007; Sutherland et al. 2016) we have also exploited the wealth of data available in TG-GATES to highlight the complex response of the NRF2 network to a range of chemical insults in PHH, and considered how perturbation of this pathway can be used in the context of understanding why certain compounds are associated with DILI in man. Our findings have important implications for the ongoing development of in vitro platforms and in silico models designed to improve the prediction of clinical DILI risk at an early stage of preclinical drug development.

## Materials and methods

### PHH isolation, culture and drug treatment

Liver tissue was obtained from the Liver Cell Lab at the Karolinska University Hospital (Huddinge, Sweden) or Aintree University Hospital (Liverpool, UK) by qualified medical staff. All patients donated tissue as part of planned liver resections for various indications (Table S1). Informed consent in writing was obtained from each patient and the study protocol conformed to the ethical guidelines of the 1975 Declaration of Helsinki. Immediately following removal from the patient, excess healthy liver parenchyma was separated from the specimen and placed in cold Eagle’s minimum essential medium, and transported to the laboratory on ice. Tissue dissociation and hepatocyte isolation were performed using a two-step collagenase perfusion procedure, as described previously (Strom et al. 1996). Only cell preparations with viability of ≥ 75% (as per Trypan blue exclusion test) were used for the experiments described here. The cell suspension was diluted in William’s medium E without phenol red, supplemented with 25 mM HEPES and 2 mM l-glutamine, pH adjusted to 7.4 (modified William’s medium E) plus 10% FBS. Cells were seeded at a density of 1 × 10^6^ cells/mL onto Type I collagen-coated plates (Beckton Dickinson, UK) and maintained at 37 °C in a 5% CO_2_ atmosphere. After 3 h, the medium was replaced with fresh modified William’s medium E not supplemented with FBS. After being allowed to adhere to the plates for a total of 16 h, untransfected cells were exposed to 1 mM diethylmaleate for 2 h, or 10 µM sulforaphane for 24 h. Compounds were dissolved in DMSO and the solvent content of the media was controlled to 0.5% in all cases.

### siRNA transfection

For siRNA transfections, siRNA duplexes targeted against either human *NRF2* (D-003755-05, subsequently referred to as siNRF2) or human *KEAP1* (D-012453-03, subsequently referred to as siKEAP1), and a scrambled non-targeting control siRNA duplex (D-001210-03, subsequently referred to as siCON), were obtained from the Dharmacon siGENOME library (Thermo Fisher Scientific, UK). Immediately prior to plating, cells from individual donors were reverse-transfected with 20 nM siRNA using Lipofectamine RNAiMAX (Life Technologies, UK) in accordance with the manufacturer’s instructions. Plated cells were maintained at 37 °C in a 5% CO_2_ atmosphere for 48 h to enable *NRF2* or *KEAP1* knockdown.

### Microarray analysis and bioinformatics

Total RNA (50 ng, free from genomic DNA) was labelled and amplified using a Low-Input Amplification Kit (Agilent, USA). Amplified Cy3-labelled RNA (600 ng) was fragmented and loaded onto SurePrint G3 Human Gene Expression 8 × 60K v2 arrays (Agilent). Following overnight hybridisation at 65 °C, the arrays were analysed at the Liverpool Centre for Genomic Research, according to the manufacturer’s instructions, using an Agilent G2505C Microarray Scanner. The data were extracted using Agilent Feature Extraction software v11.0.1.1. Differential gene expression analysis was conducted using the limma package within the R programming environment (R-Development-Core-Team 2005), enabling simultaneous comparisons between multiple treatments using design and contrast matrices via a linear regression model. To account for inter-individual differences in basal gene expression, the level of each gene was determined in siNRF2- or siKEAP1-transfected cells relative to siCON-transfected cells derived from the same donor. The significance (raw P-value) of estimated log_2_ fold changes for the contrasts was evaluated using limma function eBayes, and the impact of multiple testing was adjusted using the Benjamini and Hochberg approach (Benjamini and Hochberg 1995). Differentially expressed genes were defined as those with an adjusted *P* value < 0.05 when compared with the level in siCON-transfected cells. Ingenuity Pathway Analysis (IPA; http://www.ingenuity.com) enrichment statistics were used to reveal biological pathways perturbed in siRNA-transfected cells. Pathways represented by a single gene/protein were excluded for robustness. Gene ontology (GO) term enrichment analysis was performed using GOrilla (cbl-gorilla.cs.technion.ac.il).

### WGCNA and bioinformatics

Affymetrix HGU133-2 microarray CEL files generated from all PHH experiments were downloaded from the Open TG-GATES repository, jointly normalized using Robust Multi-array Average (RMA) using the Affy R package. Brainarray CDF (version 19) annotation were used to map probe sets to Entrez IDs (http://brainarray.mbni.med.umich.edu/Brainarray/Database/CustomCDF/genomic_curated_CDF.asp). Under this annotation, every gene is defined by a single probe set. This resulted in 17,500 probe sets, each mapping to a single gene, used for analysis. The TG-GATES repository contains 941 PHH experiments. An experiment denotes expression results for PHH treated with a given combination of compound, concentration and time compared to time-matched PHH treated with DMSO. For each experiment, log_2_ fold change values were calculated for all genes by subtracting average log2 intensity for DMSO arrays from average log_2_ intensity for treatment arrays. To identify co-expressed genes from the PHH data, we used the WGCNA R package (Zhang and Horvath 2005) and applied it to a matrix consisting of 941 rows (PHH experiments) and 17,500 columns (log_2_ fold change values for probes). We created unsigned modules (i.e. grouping together co-induced and repressed genes). The soft power-selection algorithm in WGCNA produces asymptotic curves which requires selection of an arbitrary level of agreement with a scale-free network topology (e.g. 90%). Small changes in the threshold can lead to large changes in the selected power. We reasoned that non-expressed genes in PHH, which represent ‘noise’ in microarray experiments, should not be co-expressed and, therefore, not contained within any module. Therefore, we created modules for each soft-power parameter setting of 4, 6, 8 and 10, and performed *t* tests assuming unequal variance on the category “yes/no” (indicating whether a gene is member of any module) vs. genes’ intensity in DMSO control. We selected 6 as the optimal soft-power parameter, as it maximized the t-statistic. Using WGCNA to merge similar modules (having correlation of their eigengene values ≥ 0.8), we obtained 399 modules containing 10,275 genes. As described previously (Sutherland et al. 2016, 2018), for each experiment we calculated the eigengene (or module score) which summarizes log_2_ fold change of their constituent genes. Briefly, this protocol deviates from the standard WGCNA approach in two steps: (a) when calculating a module score for a given experiment, log_2_ fold change values are scaled only (not centred and scaled) to avoid producing non-zero module scores for experiments where all underlying genes are unperturbed, and (b) the raw module score is normalized to unit variance (Z-score scaling) facilitating comparison across modules and across treatments. A normalised module score summarises the level of gene activation or repression caused by a given treatment in the context of the large collection of compound perturbations. As such, an eigengene greater than + 2.0 or smaller than − 2.0 is a large (and significant) perturbation in the context of the 941 experiments. Finally, we calculated the correlation between a module eigengene vs. underlying genes’ log_2_ fold change across the 941 experiments, indicating the extent to which the eigengene summarizes the underlying gene’s behaviour. To identify WGCNA modules that were enriched for genes found in the siRNA screen, canonical hypergeometric tests and subsequent correction for multiple comparisons (Bonferroni) were performed amongst modules that contained at least one gene identified in the siRNA screen.

### Calculation of performance indicators

An eigengene value of ≥ 2.0 was considered a positive (and significant) perturbation of a module. When a compound provoked a positive response at several time points and/or concentrations, the experiment with the highest eigengene value was used. For the combined metric, an eigengene value of ≥ 2.0 for any of the NRF2-associated modules was considered a positive perturbation. Negative control modules were selected using the following criteria: (a) comparable number of genes (6–11) to the NRF2-associated modules, (b) absence of enriched GO biological processes associated with the response to oxidative stress and related terms. After classifying the compounds based on features associated with DILI, as described in the supplementary methods, performance indicators were calculated as follows (using compounds with intrinsic biochemical reactivity as an example): sensitivity, positive perturbations by intrinsically reactive compounds divided by the total number of such compounds; specificity, 1 minus (positive perturbations by non-reactive compounds divided by the total number of such compounds); accuracy, (positive perturbations by intrinsically reactive compounds plus non-perturbations by non-reactive compounds) divided by the total number of intrinsically reactive and non-reactive compounds; positive predictive value, positive perturbations by intrinsically reactive compounds divided by the total number of positive perturbations (i.e. by intrinsically reactive and non-reactive compounds); negative predictive value, non-perturbations by non-reactive compounds divided by the total number of non-perturbations (i.e. by intrinsically reactive and non-reactive compounds). ROC curve analysis was performed using GraphPad Prism version 7.

### Supplementary methods

Details of the methods used for quantitative PCR, determination of cellular glutathione content, immunoblotting and the classification of compounds based on features associated with DILI are provided as supplementary material.

## Results

### Genetic modulation of NRF2 activity in PHH

**Fig. 1:**
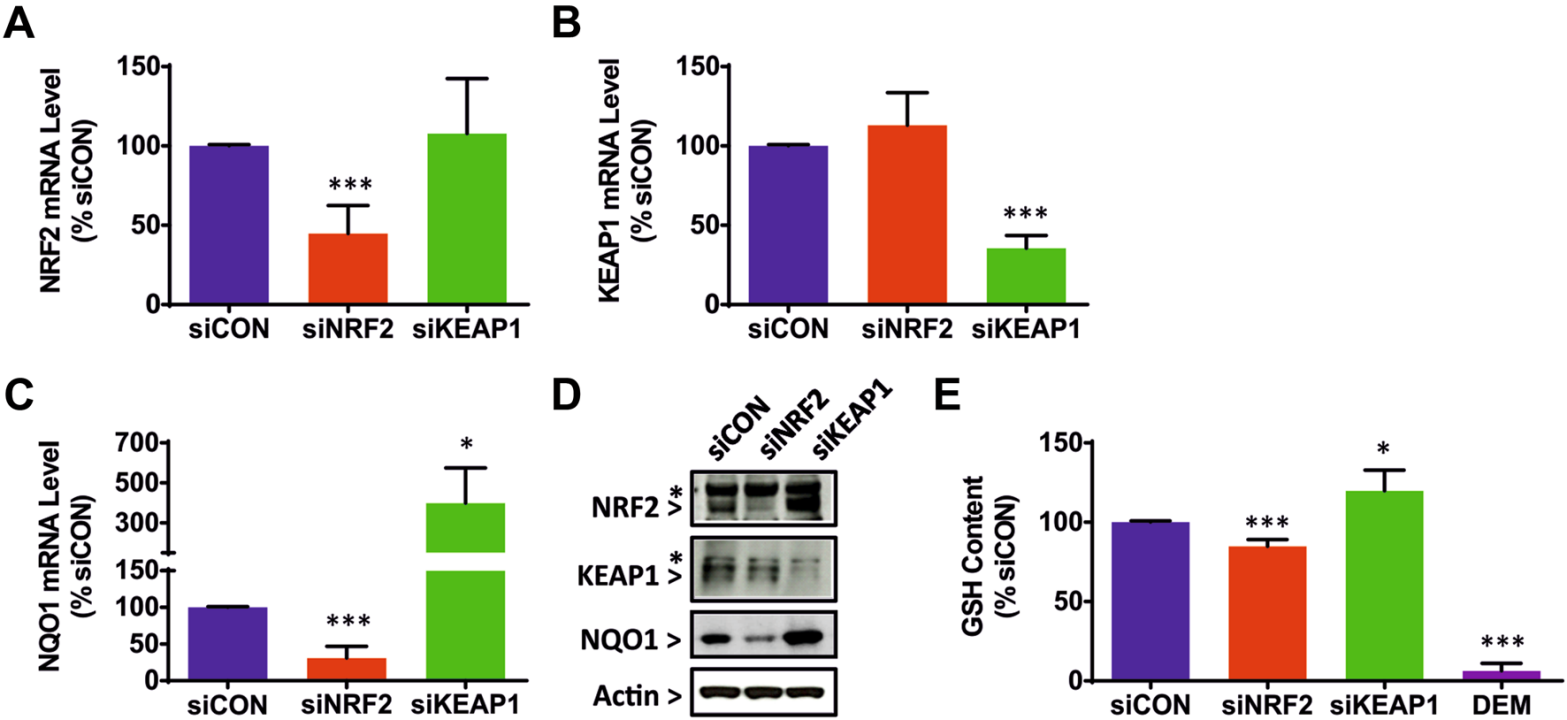
Genetic modulation of NRF2 activity in PHH. PHH isolated from four donors (Table S1A) were transfected with 20 nM siCON, siNRF2 or siKEAP1 for 48 h. **a**–**c** qPCR analysis of **a***NRF2*, **b***KEAP1* and **c***NQO1* mRNA levels in cells transfected with siCON, siNRF2 or siKEAP1. mRNA levels are normalised to *GAPDH* and expressed as a percentage of the levels in siCON-transfected cells. **d** Immunoblot analysis of NRF2, KEAP1 and NQO1 protein levels in cells transfected with siCON, siNRF2 or siKEAP1. Representative blots from one PHH donor are shown. β-Actin was probed as a loading control. *Non-specific antibody signals. **e** Quantification of GSH levels in cells transfected with siCON, siNRF2 or siKEAP1, or exposed to 1 mM diethylmaleate (DEM) for 2 h. Data represent mean + SD. of PHH from *n* = 4 donors, transfected and analysed separately. Statistical analysis of qPCR and GSH data was performed with a paired *t* test; *P ≤ 0.05 , ***P ≤ 0.001 versus siCON

In light of our interest in characterising the NRF2 transcriptional network in PHH, we first examined our previously published proteomic data sets (Bell et al. 2016; Heslop et al. 2017) to confirm that canonical NRF2 targets are expressed at comparable levels in PHH and the liver tissue from which they were isolated, and are relatively stable in cells cultured in 2D for up to 7 days, in contrast to e.g. cytochrome P450 drug metabolising enzymes which are known to exhibit a marked time-dependent decrease in expression under culture conditions (Fig. S1). These analyses justified the use of cultured PHH for investigating NRF2-regulated processes. Next, we sought to modulate NRF2 activity in PHH from four donors (Table S1A) by transfecting them with either siRNA targeting the transcription factor (siNRF2), siRNA targeting KEAP1 (siKEAP1) or a scrambled, non-targeting control duplex (siCON). Knockdown of *NRF2* and *KEAP1* mRNA in cells from each donor was confirmed by qPCR (Fig. 1a, b) and immunoblotting (Fig. 1d), and was associated with a decrease and increase, respectively, in expression of the canonical NRF2 target gene NQO1 at both mRNA and protein levels (Fig. 1c, d). In addition, significant changes in glutathione content were detected in PHH transfected with siNRF2 and siKEAP1, compared with the level in cells transfected with siCON (Fig. 1e). These data demonstrate the successful genetic modulation of NRF2 activity in PHH and the expected impact on thiol-based redox status.

### Characterisation of the NRF2 transcriptional network in PHH

**Fig. 2:**
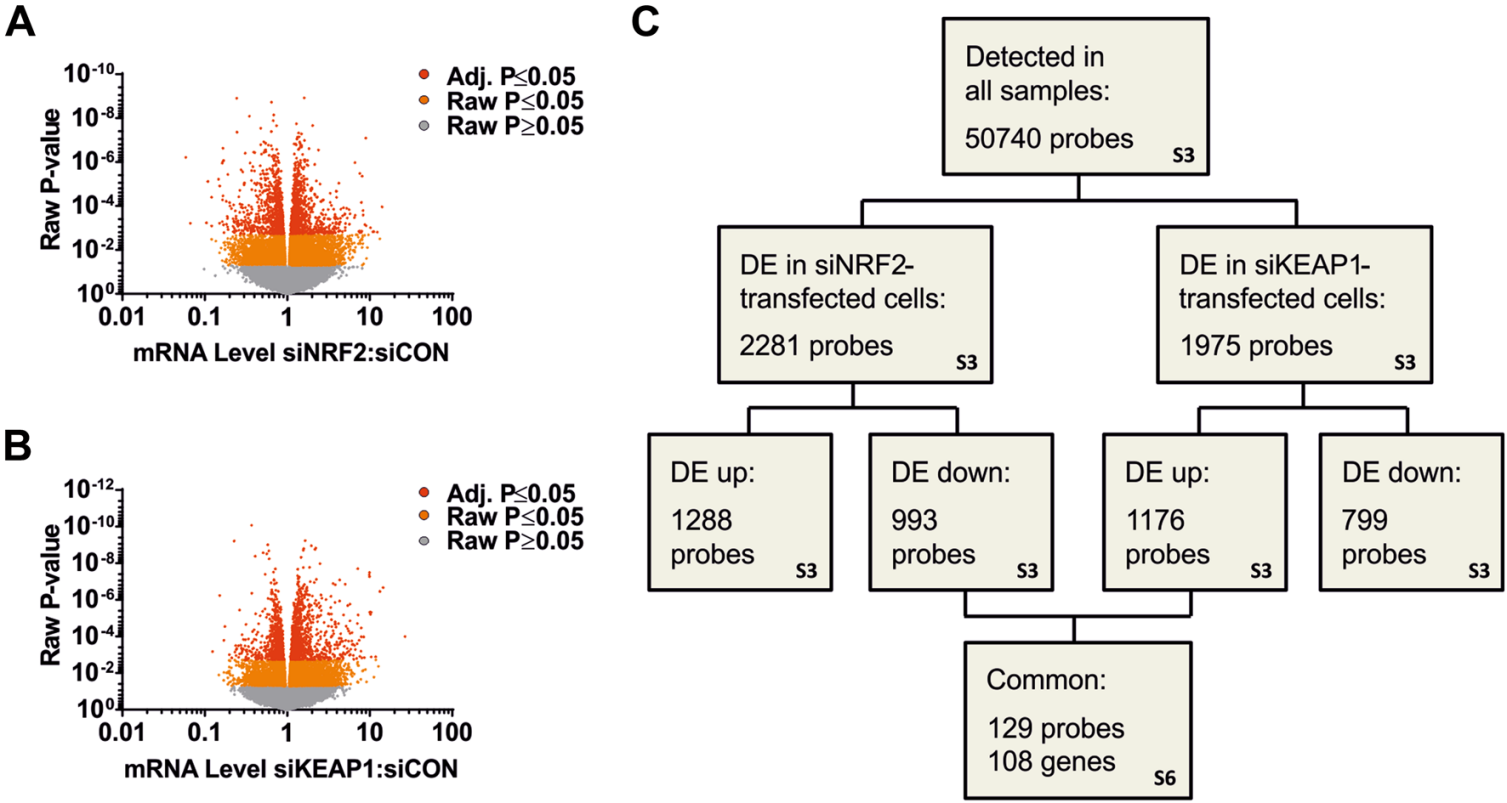
Characterisation of the NRF2 transcriptional network in PHH. Microarray analysis was performed on PHH, isolated from four donors, 48 h after transfection with siCON, siNRF2 or siKEAP1. **a, b** Volcano plots depicting differentially expressed genes in cells transfected with **a** siNRF2 and **b** siKEAP1, compared with cells from the same donor transfected with siCON. Each point represents a single gene probe, with those shaded orange (raw *P* value) and red (adjusted *P* value) found to be significantly different (*P* < 0.05) between groups. **c** Overview of microarray data sets and the strategy for the identification of genes that are highly sensitive to modulation of NRF2 activity. *DE* differentially expressed. The relevant supplementary tables are indicated in the boxes

To provide a detailed characterisation of the NRF2 network in PHH, we performed microarray analysis on RNA extracted from the cells transfected with siCON, siNRF2 or siKEAP1. Of the 50,740 probes recognised in all samples, 2281 probes (1288 up, 993 down; Fig. 2a) were differentially expressed in cells transfected with siNRF2, whilst 1975 probes (1176 up, 799 down; Fig. 2b) were differentially expressed in cells transfected with siKEAP1 (Table S3). The response of *NQO1* in the microarray analysis of cells transfected with siNRF2 or siKEAP1 showed good correlation with the targeted qPCR analysis performed on the same samples (Fig. S2), indicating the robustness of the microarray data. IPA mapping of the 993 probes that were significantly down-regulated in siNRF2-transfected cells, or the 1176 probes that were significantly up-regulated in siKEAP1-transfected cells, demonstrated that the ‘NRF2-mediated Oxidative Stress Response’ was one of the most significantly enriched pathways in both cases (Table S4), further supporting the genetic modulation of NRF2 activity in PHH. Other pathways commonly affected by both siNRF2 and siKEAP1 included ‘Xenobiotic Metabolism Signaling’, ‘Glutathione Mediated Detoxification’ and the ‘Pentose Phosphate Pathway’ (Table S4), consistent with established biological roles of NRF2. GO term enrichment analysis yielded comparable results (Table S5).

**Fig. 3:**
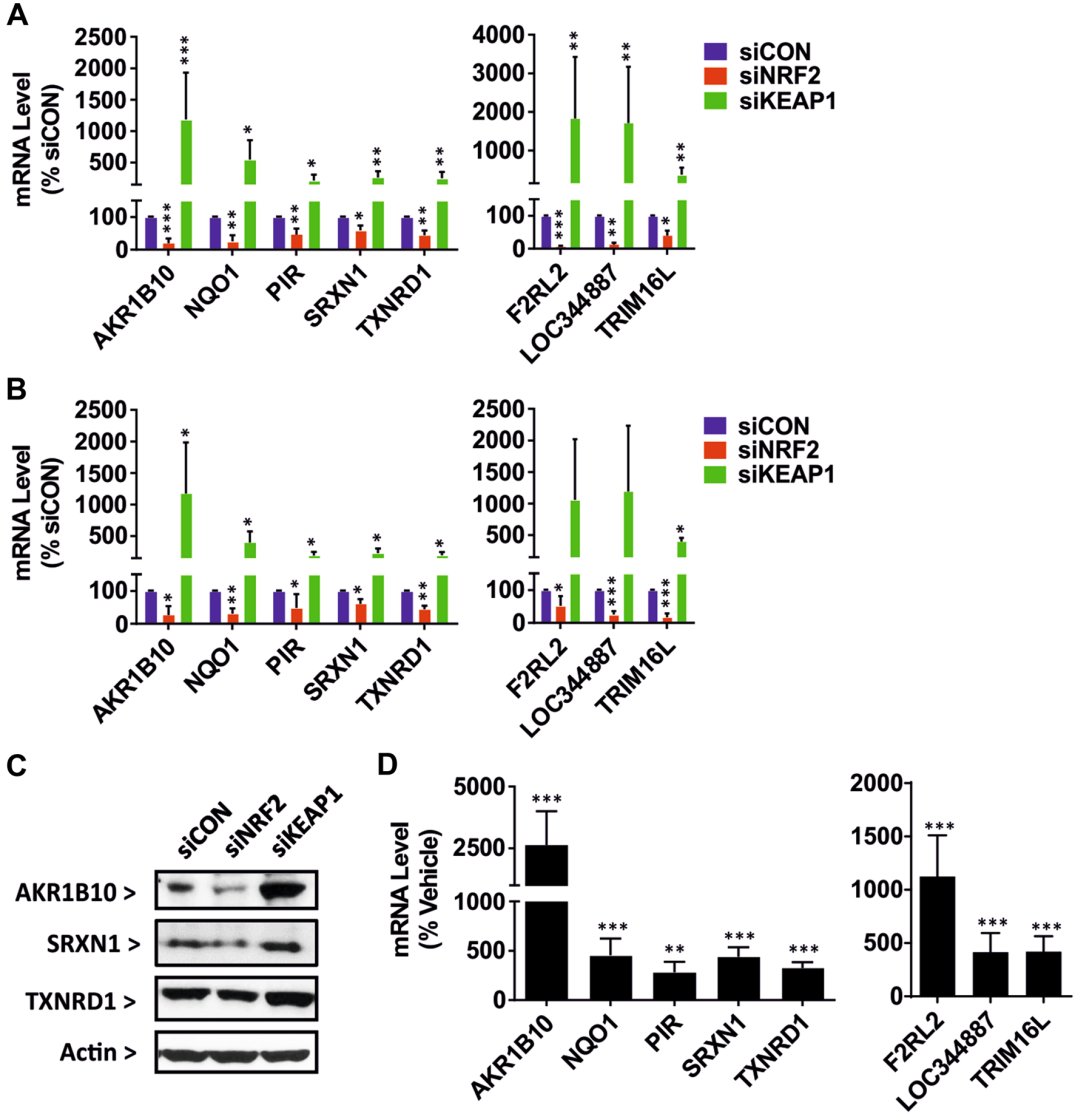
Validation of NRF2-regulated genes in PHH. **a** Microarray, **b** qPCR and **c** immunoblot determination of the indicated genes/proteins in PHH 48 h after transfection with siCON, siNRF2 or siKEAP1. **a** Note that the Agilent SurePrint G3 Human Gene Expression 8 × 60K v2 arrays contained a probe (A_23_P129903) that is homologous to sequences in the *TRIM16* and *TRIM16L* genes. **b** By qPCR, *TRIM16L*-specific primers yielded data that essentially matched that generated in the microarray, whilst *TRIM16* was barely detectable (data not shown). For qPCR, gene expression levels are normalised to *GAPDH*. **c** β-Actin was probed as a loading control. Representative blots from one PHH donor are shown. **d** qPCR determination of the indicated genes in a separate batch of PHH (Table S1B) 24 h after exposure to 10 µM sulforaphane. Data represent mean + SD of PHH from *n* = 4 donors, treated and analysed separately. Statistical analysis of qPCR data was performed with (**a, b**) a *t* test or **d** a Kruskal–Wallis (Conover-Inman) test; *P ≤ 0.05; **P ≤ 0.01;  ***P ≤ 0.001 versus siCON or DMSO

To identify genes that were most reflective of NRF2 modulation in PHH, and minimise the impact of potential off-target effects of the individual siRNAs, we focused on genes that were oppositely regulated in cells transfected with siNRF2 and siKEAP1. We identified 129 probes (representing 108 unique genes) that were significantly downregulated in siNRF2-transfected cells and upregulated in siKEAP1-transfected cells and, therefore, judged to be positively controlled by NRF2 (Fig. 2c; Table S6). Again, IPA mapping revealed that the ‘NRF2-mediated Oxidative Stress Response’ was amongst the most significantly altered pathways represented by these genes (Table S7), whilst ‘oxidation–reduction process’ was the most significantly enriched GO term (Table S8). In addition to genes known to be NRF2 targets in other cells (including *AKR1B10, NQO1, PIR, SRXN1* and *TXNRD1*), our filtering strategy also revealed several putative novel NRF2-regulated genes, including *F2RL2, LOC344887* and *TRIM16L* (Fig. 3a). We used qPCR and/or immunoblotting to confirm the altered expression of these genes in the siRNA-transfected PHH samples used to generate the microarray data (Fig. 3b, c). Furthermore, we demonstrated that these genes were upregulated in response to pharmacological activation of NRF2 by exposing PHH from four new donors (Table S1B) to sulforaphane for 24 h (Fig. 3d). Only 15 probes (representing 15 unique genes) were both significantly upregulated in siNRF2-transfected cells and downregulated in siKEAP1-transfected cells (Table S6), and thus judged to be negatively controlled by NRF2. IPA mapping identified ‘Granulocyte Adhesion and Diapedesis’ (related to the chemokines *CXCL1* and *CXCL2*) as the only significantly altered pathway represented by the 15 genes (Table S7), consistent with GO term enrichment analysis (Table S8) and the reported link between NRF2 and the inhibition of nuclear factor kB and inflammatory processes (Wardyn et al. 2015). Taken together, these data highlight established and putative novel NRF2-regulated genes, representing important physiological pathways and functions, in PHH.

### Mapping individual NRF2-regulated genes to networks using WGCNA

To further characterise the NRF2-driven gene network and provide a systems level insight into its response to chemical insult in PHH, we used WGCNA which provides a unsupervised and quantitative assessment of the interconnectedness of genes. Specifically, within a large set of gene expression data, WGCNA identifies genes that exhibit co-regulated behaviour and organizes them into defined sets of highly interconnected genes or ‘modules’, thereby reducing dimensionality and providing an unbiased interpretation of network or pathway responses (Langfelder and Horvath 2007; Zhang and Horvath 2005). We first applied WGCNA to the entire TG-GATES PHH dataset, representing expression results for 17,500 probes in cells treated with one of 158 drugs/cytokines/growth factors at up to three concentrations and three time points, compared to time-matched PHH treated with DMSO (Grinberg et al. 2014; Igarashi et al. 2015). Using this approach, we identified 399 modules comprising 10,275 genes (full details to be published separately). Of the 108 NRF2-associated genes identified in our PHH siRNA screen (Fig. 2), 66 were found to be members of 31 modules (Table S9). Amongst the remaining 42 genes, 31 (including 17 unknown/uncharacterised transcripts) were not represented in the 17,500 probes within the TG-GATES data set. Therefore, 11 NRF2-associated genes that were present in the TG-GATES data set did not meet the co-expression criteria for module membership (Table S9). Whilst the 66 NRF2-associated genes were distributed across 31 modules, five modules (namely 2, 144, 192, 224 and 325) were significantly enriched for genes identified in the siRNA screen, suggesting tight co-regulation in response to NRF2 activation (Table 1). In light of the size of module 2 (743 genes, including 15 NRF2-associated genes identified in our siRNA screen) we focussed on the remaining four modules, which comprised 6–11 genes. The hub genes for these modules (those most correlated with the eigengene value, which summarises the induction or repression of the module as a whole (Langfelder and Horvath 2007)) were *TXNRD1, CYP4F11, CD109* and *CBR3*, respectively. In addition, several of the remaining genes in the four modules were either down-regulated by siNRF2 or up-regulated by siKEAP1 in our PHH experiments (Table 1). Amongst these genes was *F2RL2* (module 325), further highlighting it as a novel NRF2-regulated gene in PHH. In keeping with this, we noted that many of the genes in the four modules have been shown by ChIP-Seq to be direct NRF2 targets in human lymphoblastoid cells (Chorley et al. 2012), whilst the modules were represented by GO terms consistent with a role in xenobiotic metabolism and/or the oxidative stress response (Table 1 and S10). These observations support the enrichment of modules 144, 192, 224 and 325 with NRF2-associated genes that exhibit consistent co-regulated behaviour in PHH exposed to a broad range of chemical insults.

**Table 1:** Features of co-expression modules statistically enriched for NRF2-regulated genes

Module	Gene	Down siNRF2	Up siKEAP1	Direct NRF2 target	Eigengene correlation	Enriched GO processes
144	*TXNRD1*	+	+	+	0.87	GO:0006081 cellular aldehyde metabolic process GO:1990748 cellular detoxification GO:0098869 cellular oxidant detoxification GO:0098754 detoxification GO:0006979 response to oxidative stress
	*SRXN1*	+	+	+	0.85	
	*GPAT3*	+			0.76	
	*CMTM8*				0.75	
	*GCLM*			+	0.74	
	*SLC6A6*		+	+	0.69	
	*PGD*	+	+	+	0.66	
	*AKR1C1*	+	+		0.59	
	*ADCK2*				0.57	
	*ADGRG7*				− 0.57	
	*DCX*		+		− 0.59	
192	*CYP4F11*		+		0.75	GO:0051187 cofactor catabolic process GO:0055114 oxidation–reduction process GO:0033015 tetrapyrrole catabolic process GO:0046149 pigment catabolic process GO:0042373 vitamin K metabolic process
	*BLVRB*		+		0.74	
	*NQO2*			+	0.73	
	*DNPEP*				0.71	
	*SLC48A1*	+	+	+	0.69	
	*GSR*	+	+	+	0.64	
	*ZDHHC9*				0.64	
	*PELI3*				0.55	
	*ZNF608*				− 0.61	
224	*CD109*		+		0.73	GO:0002573 myeloid leukocyte differentiation GO:0006108 malate metabolic process GO:0051901 positive regulation of mitochondrial depolarization GO:1904181 positive regulation of membrane depolarization GO:0055114 oxidation–reduction process
	*PIR*	+	+	+	0.73	
	*TMEM206*				0.71	
	*ME1*	+		+	0.70	
	*FOPNL*		+		0.68	
	*MLLT11*	+	+		0.67	
	*NCF2*		+		0.67	
	*LACC1*				0.66	
325	*CBR3*	+	+		0.80	GO:0006809 nitric oxide biosynthetic process GO:0046209 nitric oxide metabolic process GO:2001057 reactive nitrogen species metabolic process GO:1903409 reactive oxygen species biosynthetic process GO:0072593 reactive oxygen species metabolic process
	*EMC3*	+			0.76	
	*GLA*				0.68	
	*NQO1*	+	+	+	0.67	
	*HTATIP2*	+	+	+	0.63	
	*F2RL2*	+	+		0.55	

Genes comprising modules 144, 192, 224 and 325 are shown, along with their status in the PHH siRNA screen. Genes identified by ChIP-Seq as direct NRF2 targets (29) are indicated. Within each module, genes are ranked according to eigengene correlation, which indicates how well (hub-like) each individual gene is correlated with the module eigengene (1.0 is a perfect correlation). The top five enriched GO processes (ranked according to P-value) are shown for each module; the complete list for each module is provided in Table S10

### Response of NRF2-associated gene sets to chemical insult in PHH

**Fig. 4:**
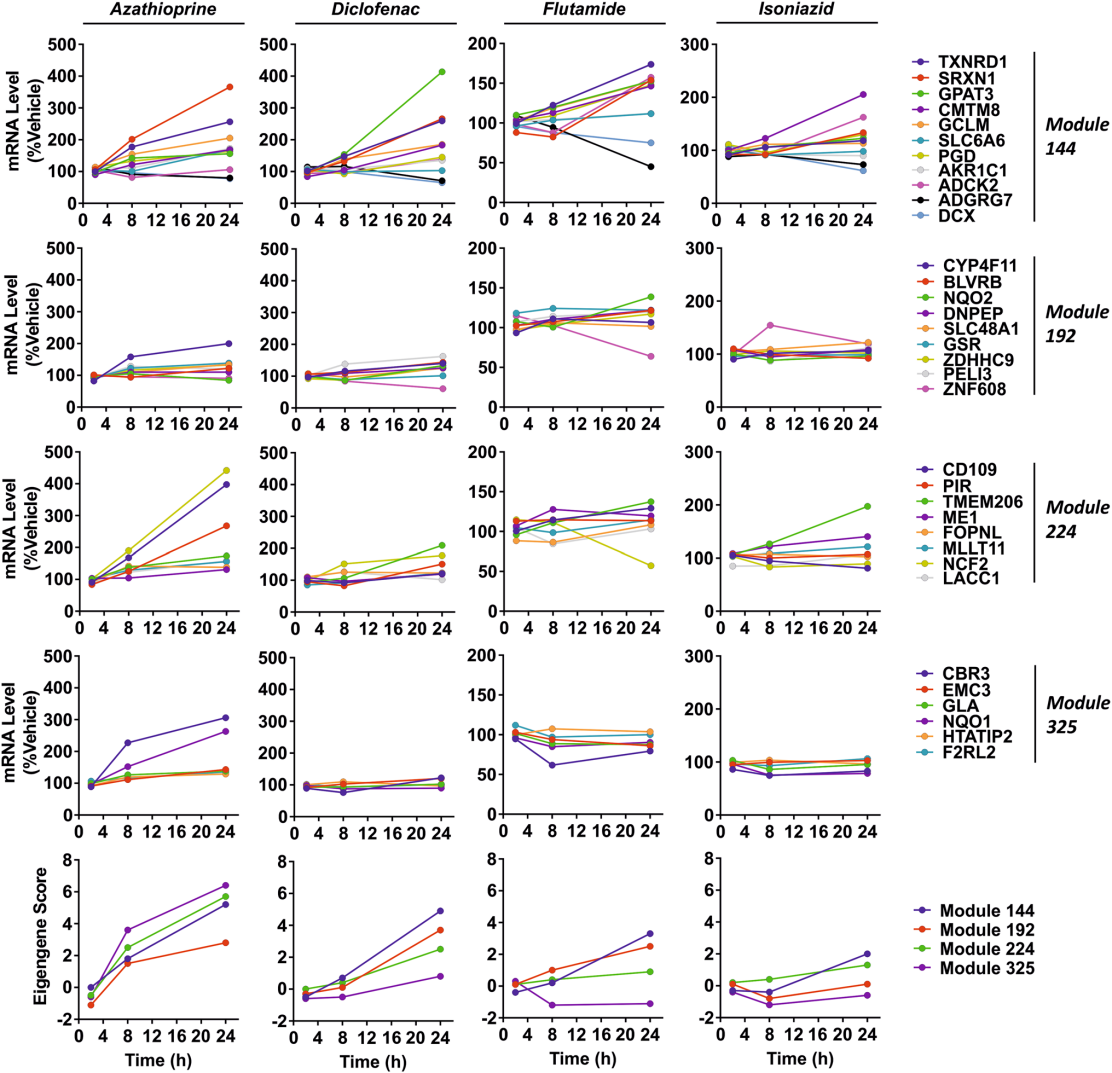
Response of NRF2-associated gene sets to chemical insult in PHH. Individual gene expression levels (% vehicle) and eigengene scores for modules 144, 192, 224 and 325 in PHH exposed to high concentrations of azathioprine (72.8 µM), diclofenac (400 µM), flutamide (50 µM) or isoniazid (10 mM) for the indicated times. All data were obtained from the TG-GATES PHH data set

Having identified modules that were enriched for NRF2-associated genes with consistent co-expression behaviour across many treatments, we sought to distinguish compounds that produced significant perturbations in these modules, and by inference the NRF2 pathway, in PHH according to the TG-GATES data set. In total 47 compounds (including 34 therapeutic drugs) caused a significant change (i.e. eigengene value ≥ 2.0) of at least one of the four modules (Table 2). Notably, modules 144 and 192 responded to more compounds (36 and 35, respectively) than modules 224 (26 compounds) and 325 (19 compounds). Indeed, some compounds were found to cause the upregulation of selected NRF2-associated modules/genes but not others. For example, the established hepatotoxins azathioprine, diclofenac, flutamide and isoniazid provoked significant changes in four, three, two and one of the NRF2-associated modules, respectively (Fig. 4). These data highlight the complex responses of NRF2-associated modules and their constituent genes to chemical insult in PHH, and highlight the value of using modules (rather than selected individual genes) to quantify network perturbation.

**Table 2:** Compounds perturbing NRF2-associated gene sets in PHH

Compound	Eigengene values for NRF2-associated modules				Therapeutic drug	DILI concern	Reactivity/ bioactivation status
	144	192	224	325			
Allyl alcohol	**4.2**	**2.3**	**2.7**	**2.2**			Reactive
Azathioprine	**5.2**	**2.8**	**5.7**	**6.4**	+	Most	Reactive
Butylated hydroxyanisole	**5.0**	**5.8**	**2.2**	**4.2**			Bioactivated
Diethyl maleate	**4.5**	**2.7**	**3.2**	**7.6**			Reactive
Furosemide	**3.2**	**3.2**	**2.1**	**3.8**	+	Ambiguous	Bioactivated
Lomustine	**2.2**	**2.7**	**2.5**	**3.8**	+	Less	Reactive
Nitrofurantoin	**6.3**	**5.1**	**3.2**	**2.5**	+	Most	Bioactivated
Nitrofurazone	**3.0**	**2.1**	**3.9**	**4.9**	+		Not BR
Phorone	**2.6**	**2.3**	**2.2**	**3.6**			Reactive
Propylthiouracil	**5.5**	**3.8**	**4.5**	**3.9**	+	Most	Reactive
Acetaminophen	**7.9**	**3.0**	1.8	**2.9**	+	Most	Bioactivated
Benzbromarone	**4.7**	**2.7**	**2.6**	0.2	+	Most	Bioactivated
Bromoethylamine	0.5	**2.4**	**4.4**	**3.6**			Bioactivated
Danazol	**2.5**	**2.5**	**2.1**	0.3	+	Most	Bioactivated
Diclofenac	**4.9**	**3.7**	**2.5**	1.5	+	Most	Bioactivated
Doxorubicin	− 0.2	**6.3**	**3.6**	**2.0**	+	Less	Bioactivated
Galactosamine	0.6	**3.7**	**2.7**	**2.8**			Unknown
Methylene dianiline	**2.9**	**2.8**	**3.6**	0.7			Bioactivated
*N*-methyl-*n*-nitrosourea	**2.2**	**3.4**	**2.4**	1.0			Reactive
Omeprazole	**7.2**	**4.7**	**3.5**	1.4	+	Less	Bioactivated
Phalloidin	0.5	**2.1**	**2.0**	**2.0**			Unknown
Phenobarbital	**2.3**	**3.1**	**4.7**	1.4	+	Less	Not BR
Tunicamycin	**2.5**	**4.0**	1.5	**4.1**			Unknown
Valproic acid	**2.1**	**2.0**	**2.8**	1.5	+	Most	Bioactivated
2,4-Dinitrophenol	2.0	**2.2**	**2.7**	0.0			Bioactivated
Adapin	**3.0**	0.7	**2.6**	0.2	+	Less	Unknown
Aflatoxin B1	− 0.7	**3.5**	**3.5**	1.2			Bioactivated
Coumarin	**3.3**	1.6	2.0	**2.6**	+		Bioactivated
Diazepam	**5.1**	**2.9**	0.1	0.1	+	Ambiguous	Bioactivated
Flutamide	**3.3**	**2.5**	0.9	0.3	+	Most	Bioactivated
Ketoconazole	**3.3**	**2.4**	1.6	0.4	+	Most	Bioactivated
Naphthyl isothiocyanate	**4.6**	**2.6**	2.0	2.0			Reactive
Nefazodone	0.1	**4.4**	**2.1**	− 0.1	+	Most	Bioactivated
Rosiglitazone maleate	**2.5**	**2.5**	0.6	− 0.3	+	Less	Bioactivated
Sulindac	**2.2**	**2.4**	− 0.3	− 1.1	+	Most	Bioactivated
Colchicine	**2.4**	1.5	− 2.0	− 1.3	+	Ambiguous	Bioactivated
Dantrolene	0.1	− 0.3	1.2	**2.9**	+	Most	Not BR
Enalapril	1.9	1.1	1.3	**3.2**	+	Less	Bioactivated
Isoniazid	**2.0**	0.1	1.3	0.0	+	Most	Bioactivated
Labetalol	**2.0**	0.9	1.6	1.1	+	Most	Not BR
Methapyrilene	**4.5**	1.2	0.2	0.5	+		Bioactivated
Methimazole	**2.5**	1.1	0.0	0.3	+	Most	Bioactivated
Moxisylyte	**2.4**	1.6	1.0	1.8	+	Most	Unknown
Papaverine	0.4	**3.2**	0.6	− 0.1	+	Most	Unknown
Perhexiline	**2.2**	0.9	1.2	0.6	+	Most	Unknown
Phenylbutazone	1.7	**2.1**	0.4	0.2	+		Bioactivated
Ranitidine	**2.3**	− 0.3	0.2	0.8	+	Less	Bioactivated

The 47 compounds causing a significant change (i.e. ≥ 2; values in bold) in the eigengene value of least one of the four NRF2-associated modules, according to the TG-GATES PHH data set. Therapeutic drugs are indicated. Bioactivation status and clinical DILI risk assignments as described in the main text

### Perturbation of NRF2-associated gene sets as an indicator of compound features associated with DILI

**Fig. 5:**
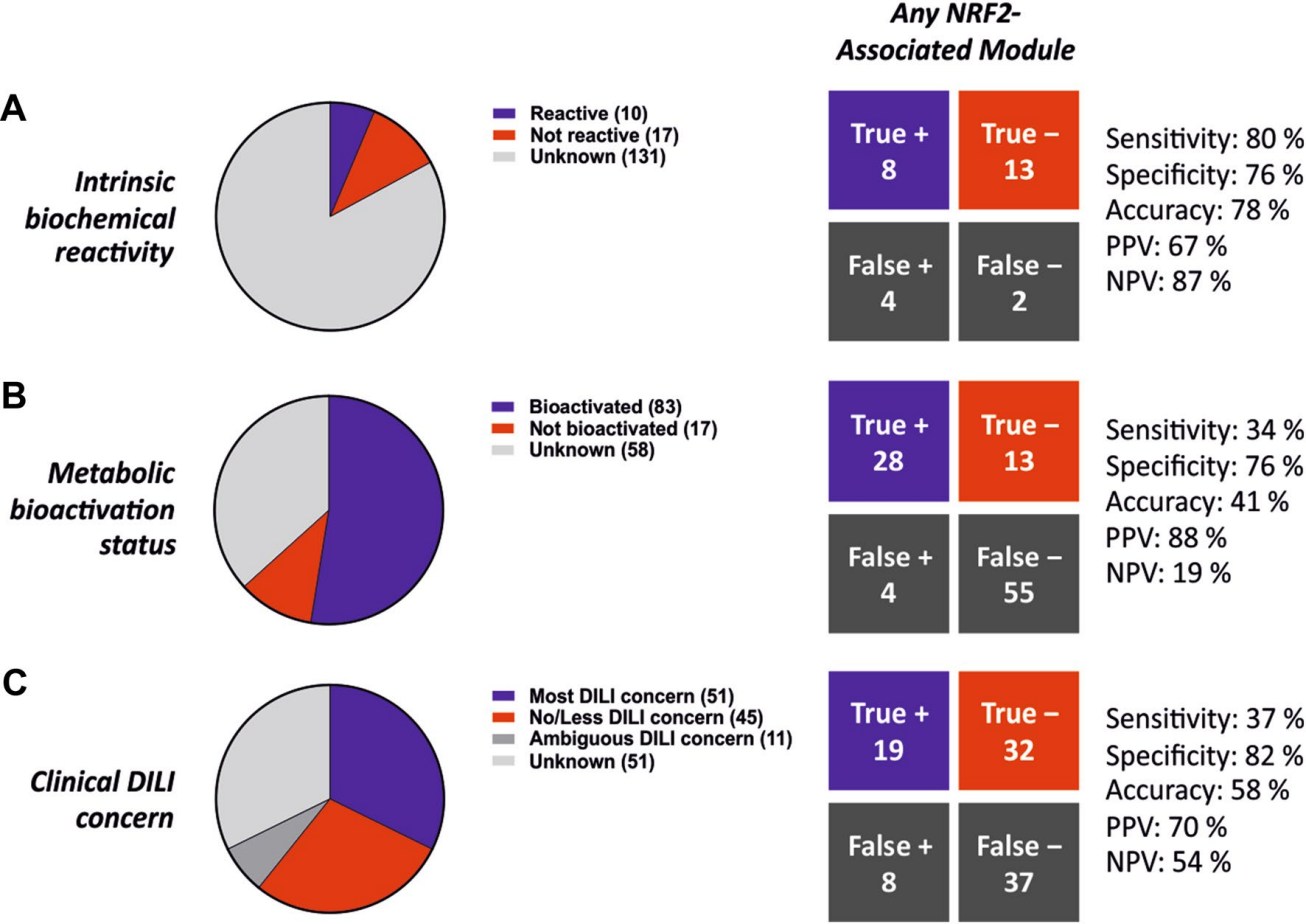
Perturbation of NRF2-associated gene sets as an indicator of compound features associated with DILI. The 158 TG-GATES compounds were classified based on **a** intrinsic biochemical reactivity, **b** metabolic bioactivation status and **c** clinical DILI concern. Compounds classified as ‘unknown’ or ‘ambiguous DILI concern’ were excluded from the relevant analyses. Based on the module eigengene values for each compound (see Tables S12–15), performance indicators were calculated (see “Experimental Procedures” for details) to determine the association between module perturbation and the respective toxicity features. See Fig. S3 for performance indicators associated with individual NRF2-associated modules. P/NPV = positive/negative predictive value

Finally, we sought to test whether the perturbation of NRF2-associated modules, and by inference the stimulation of the NRF2 transcriptional network, could be a useful indicator of clinical DILI risk and/or compound features associated with hepatotoxicity. We surveyed the literature to classify the 158 TG-GATES compounds as; (a) those with intrinsic biochemical reactivity towards cellular biomolecules (10 compounds; Fig. 5a; Table S11), (b) those bioactivated to a chemically reactive metabolite in rodent and/or human in vitro or in vivo systems (83 compounds; Fig. 5b; Table S11), and (c) those with experimental evidence for a lack of intrinsic biochemical reactivity and bioactivation (17 compounds; Table S11). To the best of our knowledge, the metabolic bioactivation status or intrinsic biochemical reactivity of the remaining 48 compounds (including seven cytokines and growth factors) has not been reported, and so these compounds were excluded from the relevant analyses. Separately, we found that 107 of the 158 compounds used in the TG-GATES PHH experiments were present in DILIrank (Chen et al. 2016), with 51 classified as ‘most DILI concern’ and 45 as ‘less/no DILI concern’ (Fig. 5c; Table S12). The 11 compounds classified as ‘ambiguous DILI concern’, along with the 51 compounds not present in DILIrank, were excluded from the analyses.

In keeping with the responsiveness of NRF2 to direct chemical stress, perturbation of the NRF2-associated modules was found to be a very good indicator of intrinsic biochemical reactivity, with relatively high sensitivities, specificities and positive/negative predictive values in each case (Fig. 5a and Fig. S3; Table S13). Importantly, the high sensitivity values associated with the perturbation of the NRF2-associated modules were not observed with a selection of similarly-sized gene sets (modules 140, 181 and 269; see Table S16 for compositions) enriched for GO processes not linked to the oxidative stress response (Fig. S4 and Table S13). NRF2 module perturbation was found to be a less sensitive indicator of metabolic bioactivation status and clinical DILI concern, with sensitivity values below 50% in all cases (Fig. 5b, c and Fig. S3; Table S14-15). However, specificity and positive predictive values remained relatively high in both contexts, suggesting that false positive detection rates should be low in these settings. These observations were supported by ROC curve analysis, with area under the curve (AUC) values above 0.8 for the prediction of intrinsic biochemical reactivity and generally below 0.6 for the prediction of metabolic bioactivation status or clinical DILI concern (Table 3 and Fig. S5). Using the two NRF2-associated modules (42 m and 320) identified in our previous WGCNA depiction of co-regulated gene sets in rat liver (Sutherland et al. 2016, 2018), we observed similar trends (relatively low sensitivity but high specificity and positive predictive values for the prediction of metabolic bioactivation status or clinical DILI concern) when analysing the associated TG-GATES *in vivo* data set (Fig. S6), indicating that our findings are relevant across species and experimental models. Taken together, these analyses show that stimulation of the NRF2 transcriptional network is a very good indicator of intrinsic biochemical reactivity/chemical stress, but is a less sensitive indicator of metabolic bioactivation status and clinical DILI concern.

**Table 3:** Area under the curve (AUC) values for NRF2-associated gene sets as indicators of compound features associated with DILI

	Module 144	Module 192	Module 224	Module 325
Intrinsic biochemical reactivity	0.818	0.806	0.800	0.847
Metabolic bioactivation status	0.556	0.629	0.572	0.535
Clinical DILI concern	0.598	0.600	0.593	0.502

Receiver operator characteristic (ROC) curve analysis was performed, based on the eigengene values of modules 144, 192, 224 and 325 for relevant compounds, to determine the association between module perturbation and the respective toxicity features (an AUC value of 1 = a perfect indicator, 0.5 = random). ROC curves are provided in Fig. S4

## Discussion

There is an urgent need to develop a more holistic pre-clinical approach to relate chemical insults to relevant biological events to better predict DILI risk in man. Given its mechanistic association with hepatotoxicity in rodents (Clarke et al. 2016), NRF2 activation has been considered a relevant biological event that could be incorporated into next-generation models affording a greater understanding of DILI, and thus an improved prediction of clinical hazard. The selection of robust marker genes representing NRF2 activation, and other stress responsive transcriptional networks, is critical to such endeavours. In this study, we have used an unbiased approach, combining siRNA gene knockdown, transcriptomics and WGCNA, to reveal the molecular landscape of the NRF2 transcriptional network in PHH and identify co-expressed genes sets with consistent responses across many compounds. In doing so, we have determined the ability of NRF2 activation to reflect compound features associated with DILI. Our findings illustrate a number of important points.

This study has demonstrated the high sensitivity of the NRF2-driven stress response in PHH to compounds with intrinsic biochemical reactivity towards cellular macromolecules, i.e. those compounds most likely to induce direct chemical stress. Whilst our findings will need to be confirmed with transcriptomics data from a larger selection of intrinsically reactive compounds, when such a resource becomes available, our observations are consistent with the canonical mechanism underpinning chemical stimulation of NRF2 signalling, whereby the transcription factor responds to agents that react with cysteine residues in KEAP1 to induce a chemopreventive phenotype at sub-toxic concentrations (Bryan et al. 2013). Notably, all of the TG-GATES PHH experiments used minimally cytotoxic concentrations of each compound. Hence, the observed NRF2-driven responses can be regarded as transcriptional adaptations to relatively minor chemical insults, further exemplifying the sensitivity of the network to direct chemical stress.

It is important to note that chemical stress may or may not result in DILI and/or others forms of overt toxicity, depending on the extent of exposure and engagement of critical cellular targets. Consistent with this, we have shown that activation of NRF2 in PHH by itself is not a sensitive predictor of clinical DILI risk. However, in light of the complexity of DILI (reflected in the range of hepatotoxicity mechanisms represented by the TG-GATES compound set) it is inconceivable that a single cell signalling pathway could adequately reflect the range of chemical and biological perturbations necessary to afford a holistic prediction of DILI risk in man. Given that NRF2 has evolved as one of several stress-responsive transcription factors that serve to protect the liver and other organs from the consequences of a range of environmental burdens, it will be important to also consider the activities of these other cellular networks to better determine the balance between benign adaptation and deleterious progression in the face of a given chemical insult, thereby improving our mechanistic understanding and ability to predict clinical DILI risk. Such efforts will be aided by the emergence of large, granular data sets encompassing measurements of several stress response pathways alongside other pertinent endpoints (Huang et al. 2016; Wink et al. 2017, 2018).

We have also shown the relatively low sensitivity of NRF2 network perturbation as an indicator of the propensity of a compound to undergo bioactivation to a reactive metabolite. Critically, reactive metabolite formation was not measured as part of the TG-GATES experiments, making it difficult to relate chemistry to biology in the same experimental setting. It is also known that reactive metabolite formation is neither necessary nor sufficient for all forms of DILI. Therefore, it is unlikely that all instances of drug bioactivation represent a genuine chemical insult, as exemplified by acetaminophen, which is safe at therapeutic doses despite 5–10% bioactivation to a reactive quinoneimine metabolite, and only toxic following overdose. NRF2 activation may, therefore, better reflect the cellular consequences of drug bioactivation, complementing chemistry-based reactive metabolite screens in the early phases of drug development, where consideration of drug exposure at relevant organs (e.g. through physiologically-based pharmacokinetic modelling) may support an improved understanding of the clinical relevance of stress response network perturbations observed in preclinical settings.

An interesting finding of our work is that the NRF2-associated modules and their constituent genes respond in a complex manner to chemical insult. Indeed, we have identified several compounds that alter the expression level of some but not all NRF2 targets. Although the detailed underlying mechanisms require further investigation, the influence of other transcription factors, cellular signalling events and post-translational modifications may underlie the ability of a set of compounds to commonly activate the NRF2 stress response yet augment the expression of different subsets of NRF2 target genes, and/or to different degrees. Using WGCNA, we have demonstrated that one approach to reducing this complexity is to consider genes as components of modules that, based on co-regulated behaviour across a large set of experiments, reflect perturbation of transcriptional networks associated with important cellular processes (Sutherland et al. 2016, 2018). Such an approach can provide an unbiased means of selecting appropriate genes as the basis for targeted analysis of stress response network perturbations. Indeed, amongst the few published investigations of NRF2 signalling responses in PHH, most have used pre-curated lists (often based on other cell types) as the basis for the selection of NRF2-regulated genes (for example, see Souza et al. 2017). However, not all of these genes may be robust markers of NRF2 activation in PHH per se. In this regard, our demonstration that *SRXN1* is oppositely regulated following siRNA knockdown of NRF2 and KEAP1 in PHH, and highly correlated with the eigengene of NRF2-associated module 144, supports our selection of this gene as a marker of NRF2 activation within a panel of HepG2 cell lines expressing green fluorescent protein-tagged components of key cellular stress response pathways, which can be used to provide a deeper understanding of the consequences for cell health of activating NRF2 and other signalling pathways in the context of different chemical toxicities (Wink et al. 2017, 2018). Interestingly, *Srxn1* is not present in Nrf2-associated modules previously defined in rat liver (Sutherland et al. 2016, 2018), further illustrating the utility of the module-based approach and context-specific nature of marker gene selection.

We and others have previously used transcriptomics and proteomics to detail the biological processes that are altered in the livers of Nrf2- or Keap1-null transgenic mice (Kitteringham et al. 2010; Walsh et al. 2014; Wu et al. 2012). In keeping with the findings of those investigations, the present study has shown that, in PHH, NRF2 regulates the expression of a battery of genes that coordinate the response to chemical and oxidative stress, the disposition of xenobiotics, and the provision of NADPH and other cellular fuels, supporting a role for NRF2 in the maintenance of normal hepatic function in man. Our study also highlights several putative novel NRF2 target genes, including *F2RL2, TRIM16L* and the pseudogene *LOC344887*. Whilst further work is needed to define the biological significance of the interaction between NRF2 and these genes, *LOC344887* (also known as *NMRAL2P*) was recently shown to respond to sulforaphane and regulate the NRF2-dependent induction of NQO1 in colon cancer cells (Johnson et al. 2017). In addition, our data highlight differences between the regulatory roles of NRF2 in the human and mouse liver, such as the high responsiveness to NRF2 modulation of members of the *AKR* family primarily in PHH, and the *GST* family predominantly in mouse liver (Kitteringham et al. 2010; Walsh et al. 2014; Wu et al. 2012). Therefore, whilst the over-arching cytoprotective function of NRF2 is conserved across mammals, it is clear that subtle differences in the regulatory roles of the transcription factor exist between species. It will be important to consider the preservation of the NRF2 network relationships described here when designing assays and in silico models intended to bridge across species. Examination of gene expression changes following non-canonical activation of the transcription factor via the glycogen synthase kinase-3 axis (Hayes et al. 2015) may further expand the landscape of the NRF2 network.

In summary, we have performed an unbiased characterisation of the NRF2 transcriptional network in PHH, used WGCNA to identify robust marker genes reflecting NRF2 activation, and shown that the perturbation of this signalling pathway is a very good indicator of the ability of a compound to cause direct chemical stress in cells. We have also shown that the activation of NRF2 signalling is a less sensitive indicator of clinical DILI concern, yet false positive detection rates are likely to be low in this setting due to the relatively high specificity and positive predictive values associated with perturbation of NRF2-associated gene sets by non-hepatotoxic compounds. In the early stages of preclinical drug toxicity assessment, high specificity assays are desirable such that safe compounds are not inappropriately terminated, whilst high sensitivity assays become more important at later stages as compounds move closer to human trials. Therefore, in this context, a positive NRF2 signal would likely merit further investigation of the toxic potential of a drug candidate. On the other hand, compounds that do not provoke a chemical stress response are unlikely to stimulate NRF2 signalling, although a lack of NRF2 signal could not guarantee safety. A better understanding of what NRF2 activation, and other relevant stress responses, can and cannot tell us about the risks associated with a given compound will contribute to the improved prediction of DILI, ensuring that drug development programs are not unnecessarily halted and supporting the progression of safe and effective new drugs into the clinic.

## Electronic supplementary material

Below is the link to the electronic supplementary material.

Supplementary material 1 (DOCX 182 KB)

Supplementary material 2 (XLSX 8313 KB)

Supplementary material 3 (XLSX 21 KB)

Supplementary material 4 (XLSX 798 KB)

Supplementary material 5 (XLSX 107 KB)

Supplementary material 6 (XLSX 16 KB)

Supplementary material 7 (XLSX 52 KB)

Supplementary material 8 (XLSX 14 KB)

Supplementary material 9 (XLSX 21 KB)

Supplementary material 10 (XLSX 36 KB)

Supplementary material 11 (XLSX 11 KB)

Supplementary material 12 (XLSX 16 KB)

Supplementary material 13 (XLSX 25 KB)

Supplementary material 14 (XLSX 25 KB)

## Abbreviations

ARE: Antioxidant response element
DILI: Drug-induced liver injury
GO: Gene ontology
IPA: Ingenuity pathway analysis
KEAP1: Kelch-like ECH-associated protein 1
NRF2: Nuclear factor erythroid 2 related factor 2
PHH: Primary human hepatocytes
WGCNA: Weighted gene co-expression analysis

## References

[CR1] Bell CC, Hendriks DF, Moro SM (2016). Characterization of primary human hepatocyte spheroids as a model system for drug-induced liver injury, liver function and disease. Sci Rep.

[CR2] Benjamini Y, Hochberg Y (1995). Controlling the false discovery rate: a practical and powerful approach to. Multiple testing. J R Stat Soc Ser B.

[CR3] Bryan HK, Olayanju A, Goldring CE, Park BK (2013). The Nrf2 cell defence pathway: Keap1-dependent and -independent mechanisms of regulation. Biochem Pharmacol.

[CR4] Chen M, Bisgin H, Tong L (2014). Toward predictive models for drug-induced liver injury in humans: are we there yet?. Biomark Med.

[CR5] Chen M, Suzuki A, Thakkar S, Yu K, Hu C, Tong W (2016). DILIrank: the largest reference drug list ranked by the risk for developing drug-induced liver injury in humans. Drug Discov Today.

[CR6] Chorley BN, Campbell MR, Wang X (2012). Identification of novel NRF2-regulated genes by ChIP-Seq: influence on retinoid X receptor alpha. Nucleic Acids Res.

[CR7] Clarke JL, Murray JB, Park BK, Copple IM (2016). Roles of Nrf2 in drug and chemical toxicity. Curr Opinion Toxicol.

[CR8] Grinberg M, Stober RM, Edlund K (2014). Toxicogenomics directory of chemically exposed human hepatocytes. Arch Toxicol.

[CR9] Hayes JD, Chowdhry S, Dinkova-Kostova AT, Sutherland C (2015). Dual regulation of transcription factor Nrf2 by Keap1 and by the combined actions of beta-TrCP and GSK-3. Biochem Soc Trans.

[CR10] Heslop JA, Rowe C, Walsh J (2017). Mechanistic evaluation of primary human hepatocyte culture using global proteomic analysis reveals a selective dedifferentiation profile. Arch Toxicol.

[CR11] Huang R, Xia M, Sakamuru S (2016). Modelling the Tox21 10 K chemical profiles for in vivo toxicity prediction and mechanism characterization. Nat Commun.

[CR12] Igarashi Y, Nakatsu N, Yamashita T (2015). Open TG-GATEs: a large-scale toxicogenomics database. Nucleic Acids Res.

[CR13] Johnson GS, Li J, Beaver LM et al (2017) A functional pseudogene, NMRAL2P, is regulated by Nrf2 and serves as a coactivator of NQO1 in sulforaphane-treated colon cancer cells. Mol Nutr Food Res 61(4) 10.1002/mnfr.201600769 10.1002/mnfr.201600769 PMC5380536 27860235

[CR14] Kitteringham NR, Abdullah A, Walsh J (2010). Proteomic analysis of Nrf2 deficient transgenic mice reveals cellular defence and lipid metabolism as primary Nrf2-dependent pathways in the liver. J Proteomics.

[CR15] Langfelder P, Horvath S (2007). Eigengene networks for studying the relationships between co-expression modules. BMC Syst Biol.

[CR16] Oshida K, Vasani N, Jones C (2015). Identification of chemical modulators of the constitutive activated receptor (CAR) in a gene expression compendium. Nucl Recept Signal.

[CR17] Oshida K, Vasani N, Thomas RS (2015). Screening a mouse liver gene expression compendium identifies modulators of the aryl hydrocarbon receptor (AhR). Toxicology.

[CR18] Oshida K, Vasani N, Thomas RS (2015). Identification of modulators of the nuclear receptor peroxisome proliferator-activated receptor alpha (PPARalpha) in a mouse liver gene expression compendium. PLoS One.

[CR19] Park BK, Boobis A, Clarke S (2011). Managing the challenge of chemically reactive metabolites in drug development. Nat Rev Drug Discov.

[CR20] Pereira CV, Nadanaciva S, Oliveira PJ, Will Y (2012). The contribution of oxidative stress to drug-induced organ toxicity and its detection in vitro and in vivo. Expert Opin Drug Metab Toxicol.

[CR21] R-Development-Core-Team (2005) R: A language and environment for statistical computing. R Foundation for Statistical Computing

[CR22] Souza TM, Kleinjans JCS, Jennen DGJ (2017). Dose and time dependencies in stress pathway responses during chemical exposure: novel insights from gene regulatory networks. Front Genet.

[CR23] Strom SC, Pisarov LA, Dorko K, Thompson MT, Schuetz JD, Schuetz EG (1996). Use of human hepatocytes to study P450 gene induction. Methods Enzymol.

[CR24] Sutherland JJ, Jolly RA, Goldstein KM, Stevens JL (2016). Assessing concordance of drug-induced transcriptional response in rodent liver and cultured hepatocytes. PLoS Comput Biol.

[CR25] Sutherland JJ, Webster YW, Willy JA (2018). Toxicogenomic module associations with pathogenesis: a network-based approach to understanding drug toxicity. Pharmacogenomics J.

[CR26] Walsh J, Jenkins RE, Wong M (2014). Identification and quantification of the basal and inducible Nrf2-dependent proteomes in mouse liver: biochemical, pharmacological and toxicological implications. J Proteomics.

[CR27] Wardyn JD, Ponsford AH, Sanderson CM (2015). Dissecting molecular cross-talk between Nrf2 and NF-kappaB response pathways. Biochem Soc Trans.

[CR28] Wink S, Hiemstra S, Huppelschoten S (2014). Quantitative high content imaging of cellular adaptive stress response pathways in toxicity for chemical safety assessment. Chem Res Toxicol.

[CR29] Wink S, Hiemstra S, Herpers B, van de Water B (2017). High-content imaging-based BAC-GFP toxicity pathway reporters to assess chemical adversity liabilities. Arch Toxicol.

[CR30] Wink S, Hiemstra SW, Huppelschoten S, Klip JE, van de Water B (2018). Dynamic imaging of adaptive stress response pathway activation for prediction of drug induced liver injury. Arch Toxicol.

[CR31] Wu KC, Cui JY, Klaassen CD (2012). Effect of graded Nrf2 activation on phase-I and -II drug metabolizing enzymes and transporters in mouse liver. PLoS One.

[CR32] Zhang B, Horvath S (2005). A general framework for weighted gene co-expression network analysis. Stat Appl Genet Mol Biol.

