# Supplementary material for: Characterisation of the NRF2 transcriptional network and its response to chemical insult in primary human hepatocytes: implications for prediction of drug-induced liver injury
Source: Arch Toxicol. 2018 Nov 13;93(2):385–99. doi: 10.1007/s00204-018-2354-1 (PMC6373176; doi:10.1007/s00204-018-2354-1)
Supplement: Supplementary file 1 — Supplementary material 1 (DOCX 182 KB) [file 204_2018_2354_MOESM1_ESM.docx]

*Supplementary Material*

**Characterisation of the NRF2 transcriptional network and its response to chemical insult in primary human hepatocytes: Implications for prediction of drug-induced liver injury**

Ian M. Copple, Wouter den Hollander, Giulia Callegaro, Fiona E. Mutter, James L. Maggs, Amy L. Schofield, Lucille Rainbow, Yongxiang Fang, Jeffrey J. Sutherland, Ewa C. Ellis, Magnus Ingelman-Sundberg, Stephen W. Fenwick, Christopher E. Goldring, Bob van de Water, James L. Stevens, B. Kevin Park.

**Supplementary Methods**

*Quantitative PCR* – Total RNA was isolated using QIAzol lysis reagent or an RNeasy Mini kit (Qiagen, UK). cDNA was synthesised using the SuperScript III Reverse Transcription System (Life Technologies). Quantitative real-time PCR (qPCR) was performed as previously described (Copple et al., 2010, J Biol Chem, 285; 16782-16788). Gene-specific primers are detailed in Table S2.

*Determination of cellular glutathione content* – Total cellular glutathione content was quantified as previously described (Copple et al., 2010, J Biol Chem, 285; 16782-16788). Cells were exposed to 1 mM diethylmaleate for 1 h as a positive control for glutathione depletion.

*Immunoblotting –* Cells were lysed in radioimmunoprecipitation buffer, and subjected to immunoblot analysis as previously described (Copple et al., 2010, J Biol Chem, 285; 16782-16788). Antibodies raised against AKR1B10 (ab62218), KEAP1 (sc13246), NQO1 (ab2346), NRF2 (ab62352), TXNRD1 (ab124954) and SRXN1 (ab92298) were purchased from Abcam (UK) or Santa Cruz Biotechnology (Germany). Immunoreactive band volumes were quantified using TotalLab 100 software (Nonlinear Dynamics, UK) and normalised to the loading control, β-actin (ab6276).

*Classification of compounds based on features associated with toxicity –* The 158 compounds used in PHH experiments by the TG-GATES consortium were sub-categorised into three classes: (a) those with intrinsic biochemical reactivity capable of undergoing direct covalent reaction with proteins, DNA, lipids or glutathione, (b) those with evidence for formation of a chemically reactive metabolite in human and/or rodent sub-cellular, cellular and/or whole animal models, and (c) those with experimental evidence for a lack of direct/bioactivation-dependent reaction with the above biomolecules. All remaining compounds (i.e. those without reported evidence of intrinsic biochemical reactivity or bioactivation) were classed as unknowns and excluded from relevant analyses. Intrinsically reactive compounds that also undergo bioactivation (allyl alcohol, azathioprine, lomustine and propylthiouracil) were classed as intrinsically reactive. Compounds were not classed as either intrinsically reactive or bioactivated where only evidence of a biological response (e.g. upregulation of antioxidant genes) or depletion of glutathione (without direct evidence of the formation of adducts) was available. See supplementary material for additional details of the literature search methodology. In order to test the association of gene/module perturbations with clinical DILI risk, compounds were classified as ‘most DILI concern’ or ‘no/less DILI concern’ according to the DILIrank annotation of 1036 FDA-approved drugs (1).

*Definitions of bioactivation and compound assignment types –* The qualitative assessment of whether there is evidence that a listed compound undergoes bioactivation in human and/or rodent models (sub**-**cellular, cellular, tissue slice and/or whole animal) was based on an essentially biochemical definition of bioactivation. Therefore it included specific chemical or biochemical evidence of covalent reactions with biomolecules (proteins, DNA, lipids and glutathione; commonly and practically referred to as ‘irreversible binding’ in the case of binding to sub**-**cellular, whole**-**cell and whole**-**organ proteins) and excluded circumstantial evidence based on biological responses to chemically reactive species; in particular, increases in the expression of antioxidant genes (see, for example, Takakusa *et al.*, 2008, Drug Metab Dispos, 36; 816-823). Many reports of ‘mechanism**-**based’ (time/concentration**/**NADPH- dependent) inhibition of cytochrome P450 enzymes were treated cautiously; such mechanism-based inhibition is now known to be a complex, multifarious phenomenon (see, for example, Hong *et al.*, 2016, Mol Pharmacol, 89; 1-13) that requires careful deconstruction before it can be taken confidently as evidence of reactive metabolite formation. Depletion of glutathione, in isolated hepatocytes or whole liver was not taken as evidence that in itself warranted the assignment of a compound as bioactivated. The mechanisms of glutathione depletion by foreign compounds are numerous, and the correlative relationships of glutathione depletion to the ‘gold standards’ of irreversible binding and thioether metabolite formation have not been reported.

Four types of compound assignment were made:

1. Intrinsically reactive (10)
2. Bioactivated (83)
3. No evidence of bioactivation (17)
4. Bioactivation assessment not reported (48)

Most of the bioactivated compounds are known to undergo bioactivation in more than one system. Data conflicts are indicated; as in the case of ibuprofen. Uncertain assignments, such as enalapril, are also indicated. No assessment of bioactivation could be found in many cases (30 % of the 158 compounds). Some compounds, such as caffeine, are frequently used as negative controls for bioactivation. Whilst many of the compounds with unreported/unknown bioactivation status (e.g. acarbose, dexamethasone) are likely negative for bioactivation, others (e.g. adapin [doxepin], chlorpheniramine) are by obvious structural analogy clear bioactivation candidates. Therefore, all such compounds have been assigned as not reported to avoid biased/incorrect classification.

*Use of major published compound sub-sets –* Firstly, the following six published sub**-**sets of drugs assessed for bioactivation (irreversible binding and/or glutathione adduct formation, *in vitro* and/or *in vivo*, in human and/or rat models) were searched.

- Bauman JN, Kelly JM, Tripathy S, Zhao SX, Lam WW, Kalgutkar AS and Obach RS (2009) Can in vitro metabolism-dependent covalent binding data distinguish hepatotoxic from nonhepatotoxic drugs? An analysis using human hepatocytes and liver S-9 fraction. *Chem Res Toxicol* 22:332-340.
- Masubuchi N, Makino C and Murayama N (2007) Prediction of in vivo potential for metabolic activation of drugs into chemically reactive intermediate: correlation of in vitro and in vivo generation of reactive intermediates and in vitro glutathione conjugate formation in rats and humans. *Chem Res Toxicol* 20:455-464.
- Nakayama S, Atsumi R, Takakusa H, Kobayashi Y, Kurihara A, Nagai Y, Nakai D and Okazaki O (2009) A zone classification system for risk assessment of idiosyncratic drug toxicity using daily dose and covalent binding. *Drug Metab Dispos* 37:1970-1977.
- Obach RS, Kalgutkar AS, Soglia JR and Zhao SX (2008) Can in vitro metabolism-dependent covalent binding data in liver microsomes distinguish hepatotoxic from nonhepatotoxic drugs? An analysis of 18 drugs with consideration of intrinsic clearance and daily dose. *Chem Res Toxicol* 21:1814-1822.
- Thompson RA, Isin EM, Li Y, Weidolf L, Page K, Wilson I, Swallow S, Middleton B, Stahl S, Foster AJ, Dolgos H, Weaver R and Kenna JG (2012) In vitro approach to assess the potential for risk of idiosyncratic adverse reactions caused by candidate drugs. *Chem Res Toxicol* 25:1616-1632.
- Usui T, Mise M, Hashizume T, Yabuki M and Komuro S (2009) Evaluation of the potential for drug-induced liver injury based on in vitro covalent binding to human liver proteins. *Drug Metab Dispos* 37:2383-2392.

Those searches found 34 bioactivated compounds, representing 40 % of the final total of compounds shown to undergo metabolic bioactivation. The searches also revealed 15 compounds (‘negatives’) for which either no or only marginal evidence of bioactivation, such as exceptionally low levels of irreversible binding, had been obtained.

*Search methodology for compounds not found in the major published sub-sets –* The primary database searched was PubMed. The following search terms were used singly with the name of the compound:

1. bioactivation
2. reactive metabolite(s)
3. covalent binding
4. irreversible binding
5. glutathione

If none of those five searches yielded evidence of bioactivation, a wider investigation of the compound’s metabolism and metabolites was performed. However, the secondary searches were generally unproductive. In exceptional cases, as with trimethadione, the secondary search yielded evidence of bioactivation by purified human enzymes. Isolated instances of bioactivation by non**-**mammalian enzymes, such as plant peroxidases, were ignored.

In most instances, the compounds that were categorized as intrinsically reactive, such as the nitroso compounds, were self**-**evidently of that type. The nine drugs known to be metabolized to acyl glucuronides were categorized as bioactivated because of the well**-**known electrophilicity of those conjugates (Stachulski *et al.*, 2006, J Med Chem, 49; 6931-6945). Any compound that is a metabolic precursor of a substrate for bioactivation, such as azathioprine, was categorized as bioactivated.

| **Donor ID** | **Sex** | **Age** | **Indication** | **Cell Viability (%)** |
| --- | --- | --- | --- | --- |
| PHH 1 | Male | 59 | Cirrhosis | 81 |
| PHH 3 | Female | 74 | Hepatocellular carcinoma | 90 |
| PHH 4 | Female | 50 | Colorectal liver metastases | 79 |
| PHH 6 | Male | 15 | Crigler-Najjar syndrome type I | 76 |

**Table S1A – Details of patients donating liver tissue for isolation of the PHH used for microarray, qPCR and immunoblot analyses of the effects of genetic modulation of NRF2 with siRNA.**

| **Donor ID** | **Sex** | **Age** | **Indication** | **Cell Viability (%)** |
| --- | --- | --- | --- | --- |
| PHH 200 | Male | 65 | Hepatocellular carcinoma | 97 |
| PHH 203 | Male | 36 | Colorectal liver metastasis | 94 |
| PHH 206 | Male | 65 | Colorectal liver metastasis | 89 |
| PHH 207 | Female | 69 | Colorectal liver metastasis | 92 |

**Table S1B – Details of patients donating liver tissue for isolation of the PHH used for qPCR analysis of the effects of pharmacological modulation of NRF2 with sulforaphane.**

| **Gene** | **Primer** | **Sequence** |
| --- | --- | --- |
| Human *AKR1B10* | Sense | 5’- GGA GGG CCT GTA ACG TGT TG -3’ |
|  | Antisense | 5’- CAG CAC CTC GAT TCT CGT CT -3’ |
| Human *F2RL2* | Sense | 5’- ACT GAG GTG AAA TTG TGC TCC -3’ |
|  | Antisense | 5’- TTT CCA TGC CAC TCT GAC AA -3’ |
| Human *KEAP1* | Sense | 5’- CAG ATT GGC TGT GTG GAG TT -3’ |
|  | Antisense | 5’- GCT GTT CGC AGT CGT ACT TG -3’ |
| Human *LOC344887* | Sense | 5’- CTG GAG AAC GTC AAG CGA CT -3’ |
|  | Antisense | 5’- GGG ATG CCA ATG GAC CAG AA -3’ |
| Human *NQO1* | Sense | 5’- AGG ACC CTT CCG GAG TAA GAA -3’ |
|  | Antisense | 5’- TGG AAG CCA CAG AAA TGC AGA -3’ |
| Human *NRF2* | Sense | 5’- GAG AGC CCA GTC TTC ATT GC -3’ |
|  | Antisense | 5’- TTG GCT TCT GGA CTT GGA AC -3’ |
| Human *PIR* | Sense | 5’- CTA CCG CAG CGT GAG TAC C -3’ |
|  | Antisense | 5’- TAA CTC GGG TCT GCC AAT GC -3’ |
| Human *SRXN1* | Sense | 5’- GAT CCG GGA GGA CCC AGA CA -3’ |
|  | Antisense | 5’- CAA GGA GGC TGC TAC TGC AA -3’ |
| Human *TRIM16L* | Sense | 5’- GTC TCC TTC TGT GTG CAT TGG -3’ |
|  | Antisense | 5’- ACA CCG ACA CCA GAA CAG A -3’ |
| Human *TXNRD1* | Sense | 5’- ATG TCA TGT GAG GAC GGT CGG -3’ |
|  | Antisense | 5’- GGC CGC CTA TCT TTC TCT GTT -3’ |
| Human *GAPDH* | Sense | 5’- GGC CTC CAA GGA GTA AGA CC -3’ |
|  | Antisense | 5’- AGG GGT CTA CAT GGC AAC TG -3’ |

**Table S2 – Primers used for qPCR analysis of gene expression.**

**Supplementary Results**

**Fig. S1 - Relative stability of canonical NRF2 targets in cultured PHH.** Expression levels of the indicated NRF2-regulated proteins, plus two cytochrome P450 enzymes exhibiting marked decrease in expression over time, according to the indicated comparisons. Data for the comparison of freshly isolated cells and matched liver tissue was extracted from Bell et al. (2), whilst data used for the comparisons of freshly isolated cells with those cultured in 2D for up to 168 h was extracted from Heslop et al. (3). In each case, data represent mean + S.D. of n=4 individual donors.


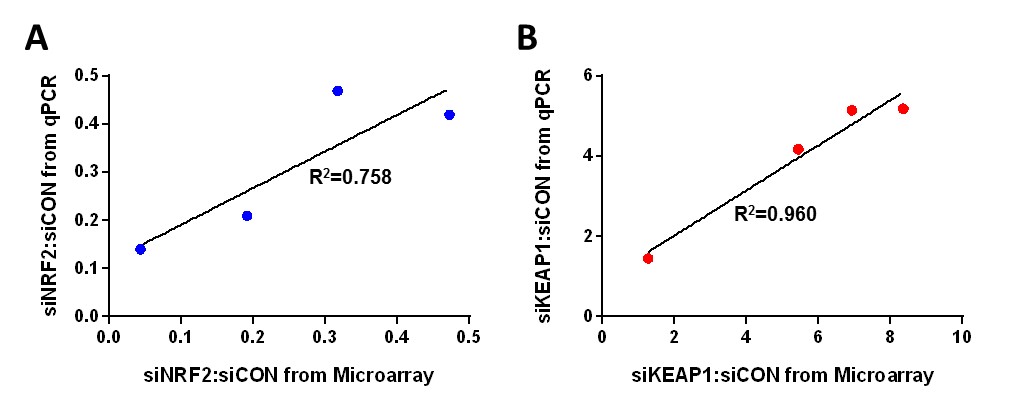


**Fig. S2 - Correlation of microarray and qPCR quantifications of NQO1 mRNA in PHH transfected with (A) siNRF2 or (B) siKEAP1.** Each data point represents cells obtained from a different donor.

**Fig. S3 - Perturbation of human NRF2-associated gene sets as an indicator of compound features associated with DILI.** The 158 TG-GATES compounds were classified based on (A) clinical DILI concern, (B) metabolic bioactivation status or (C) intrinsic biochemical reactivity. Compounds classified as ‘ambiguous DILI concern’ or ‘unknown’ were excluded from the relevant analyses. Based on the module eigengene values for each compound (see Tables S19-21), performance indicators were calculated (see Experimental Procedures for details) to determine the association between perturbation of individual NRF2-associated modules and the respective toxicity features. P/NPV = positive/negative predictive value.

**Fig. S4 – Perturbation of human gene sets not associated with the oxidative stress response as an indicator of compound features associated with DILI.** The 158 TG-GATES compounds were classified based on (A) intrinsic biochemical reactivity, (B) metabolic bioactivation status and (C) clinical DILI concern, as previously. Based on the module eigengene values for each compound (see Tables S19-21), performance indicators were calculated (see Experimental Procedures for details) to determine the association between module perturbation and the respective toxicity features.

|  |  | ***PHH siRNA Screen*** | |  |  |  |
| --- | --- | --- | --- | --- | --- | --- |
| **Module** | **Gene** | **Down siNRF2** | **Up siKEAP1** | **Eigengene Correlation** | **Enriched GO Processes** | |
| 140 | MINPP1 | + |  | 0.75 | GO:0033385  geranylgeranyl diphosphate metabolic process  GO:0033386  geranylgeranyl diphosphate biosynthetic process | |
|  | DHX40 |  |  | 0.74 |  |  |
|  | DNAJC24 |  |  | 0.71 |  |  |
|  | RPRD1A |  |  | 0.66 |  |  |
|  | TTC14 |  |  | 0.65 |  |  |
|  | GGPS1 |  |  | 0.62 |  |  |
|  | CGGBP1 |  |  | 0.60 |  |  |
|  | GSKIP |  |  | 0.58 |  |  |
|  | KIZ |  |  | 0.54 |  |  |
|  | MON2 |  |  | 0.52 |  |  |
|  | RASSF7 |  |  | -0.66 |  |  |
| 181 | POLR2I |  |  | 0.79 | GO:0001192  maintenance of transcriptional fidelity during DNA-templated transcription elongation  GO:0001193  maintenance of transcriptional fidelity during DNA-templated transcription elongation from RNA polymerase II promoter | |
|  | MRPL52 |  |  | 0.78 |  |  |
|  | TSTA3 |  |  | 0.77 |  |  |
|  | TAF10 |  |  | 0.75 |  |  |
|  | CSNK2B |  |  | 0.68 |  |  |
|  | MEA1 |  |  | 0.68 |  |  |
|  | C12orf10 |  |  | 0.67 |  |  |
|  | MYL6B |  |  | 0.65 |  |  |
|  | FAM98C |  |  | 0.61 |  |  |
| 269 | ZRANB2 |  | + | 0.78 | GO:0097036  regulation of plasma membrane sterol distribution | |
|  | COA5 |  |  | 0.76 |  |  |
|  | ARV1 |  | + | 0.75 |  |  |
|  | TMEM167A |  |  | 0.75 |  |  |
|  | ZNF226 |  |  | 0.64 |  |  |
|  | LINC00667 |  |  | 0.62 |  |  |
|  | BORCS7 |  |  | 0.60 |  |  |

**Table S16 – Features of co-expression modules not enriched for NRF2-regulated genes.** Genes comprising modules 140, 181 and 269 are shown, along with their status in the PHH siRNA screen. Within each module, genes are ranked according to eigengene correlation.

**Fig. S5 – ROC curve analysis of NRF2-associated genes sets as indicators of compound features associated with DILI.** ROC curve analysis was performed, based on the eigengene values of modules 144, 192, 224 and 325 for relevant compounds (see Tables S19-21), to determine the association between module perturbation and the respective toxicity features. AUC = area under the curve (1 = perfect indicator, 0.5 = random).

**Fig. S6 – Perturbation of rat NRF2-associated gene sets as an indicator of compound features associated with DILI.** The 158 TG-GATES compounds were classified based on (A) clinical DILI concern, (B) metabolic bioactivation status or (C) intrinsic biochemical reactivity, as previously. Compounds classified as ‘ambiguous DILI concern’ or ‘unknown’ were excluded from the relevant analyses. Rat modules were defined previously (doi:10.1038/tpj.2017.17). Based on the module eigengene values for each compound, performance indicators were calculated (see Experimental Procedures for details) to determine the association between perturbation of individual NRF2-associated modules and the respective toxicity features.

**Supplementary Tables Provided as Separate Files**

Table S3 – Microarray data for all probes, including those differentially expressed in PHH transfected with siNRF2 or siKEAP1.

Table S4 – Ingenuity Pathway Analysis of genes significantly up/down -regulated in PHH transfected with siNRF2 or siKEAP1.

Table S5 – Gene ontology term enrichment analysis of genes significantly up/down -regulated in PHH transfected with siNRF2 or siKEAP1.

Table S6 – Microarray data for probes oppositely regulated in PHH transfected with siNRF2 and siKEAP1.

Table S7 – Ingenuity Pathway Analysis of genes oppositely regulated in PHH transfected with siNRF2 and siKEAP1.

Table S8 – Gene ontology term enrichment analysis of genes oppositely regulated in PHH transfected with siNRF2 and siKEAP1.

Table S9 – WGCNA co-expression modules containing NRF2-regulated genes identified in the PHH siRNA screen.

Table S10 – Gene ontology term enrichment analysis of genes comprising WGCNA co-expression modules 144, 192, 224 and 325.

Table S11 – Classification of TG-GATES compounds based on evidence for intrinsic biochemical reactivity or propensity to form a reactive metabolite in relevant human or rodent systems.

Table S12 – Classification of TG-GATES compounds based on clinical DILI concern, according to DILIrank.

Table S13 – Performance indicators for NRF2-associated modules as indicators of intrinsic biochemical reactivity.

Table S14 – Performance indicators for NRF2-associated modules as indicators of metabolic bioactivation status.

Table S15 – Performance indicators for NRF2-associated modules as indicators of clinical DILI concern.

**Supplementary References**

1. Chen M, Suzuki A, Thakkar S, Yu K, Hu C, Tong W. DILIrank: the largest reference drug list ranked by the risk for developing drug-induced liver injury in humans. Drug Discov Today 2016;21:648-653.

2. Bell CC, Hendriks DF, Moro SM, Ellis E, Walsh J, Renblom A, Fredriksson Puigvert L, et al. Characterization of primary human hepatocyte spheroids as a model system for drug-induced liver injury, liver function and disease. Sci Rep 2016;6:25187.

3. Heslop JA, Rowe C, Walsh J, Sison-Young R, Jenkins R, Kamalian L, Kia R, et al. Mechanistic evaluation of primary human hepatocyte culture using global proteomic analysis reveals a selective dedifferentiation profile. Arch Toxicol 2017;91:439-452.
